# The Effects of Adding Probiotic, Alone and in Combination With Zinc, to Routine Treatment on Recurrence of Hepatic Encephalopathy, Quality of Life, and Sleep Quality in Patients With Cirrhosis: An Open‐Label Randomized Controlled Trial

**DOI:** 10.1002/fsn3.4636

**Published:** 2024-12-05

**Authors:** Leila Amooyi, Leila Alizadeh, Parvin Sarbakhsh, Sara Shojaei‐Zarghani, Afshin Gharekhani

**Affiliations:** ^1^ Student Research Committee, Faculty of Pharmacy Tabriz University of Medical Sciences Tabriz Iran; ^2^ Liver and Gastrointestinal Diseases Research Center Tabriz University of Medical Sciences Tabriz Iran; ^3^ Department of Biostatistics and Epidemiology, Faculty of Health Tabriz University of Medical Sciences Tabriz Iran; ^4^ Colorectal Research Center Shiraz University of Medical Sciences Shiraz Iran; ^5^ Department of Clinical Pharmacy (Pharmacotherapy), Drug Applied Research Center Tabriz University of Medical Sciences Tabriz Iran

**Keywords:** cirrhosis, hepatic encephalopathy, lactulose, probiotic, zinc

## Abstract

Overt hepatic encephalopathy (OHE) is a common complication of decompensated cirrhosis. This study aimed to assess the effects of probiotic, alone and in combination with zinc, on OHE recurrence, Model for End‐stage Liver Disease (MELD) score, ammonia level, health‐related quality of life (HRQoL), and sleep quality in patients with cirrhosis. We performed an open‐label randomized controlled trial on patients with decompensated cirrhosis with a previous history of OHE. Eligible patients (*n* = 45) were divided randomly into three equal groups to receive 30–45 mL lactulose syrup (2–4 times/day), lactulose and probiotic (4.5 × 10^11^ CFU of bacteria, 2 times/day), or a combination of lactulose, probiotic, and zinc (25 mg) for 3 months. In this study, probiotic, alone or in combination with zinc, had no effect on OHE recurrence, ammonia levels, and MELD score. Mental aspects and total score of HRQoL were improved following probiotic and its combination with zinc, even after adjustment for baseline values, age, and sex. However, physical aspects of HRQoL and sleep quality were only improved by probiotic and zinc combination compared to the lactulose alone. Our findings showed improved HRQoL after treatment with probiotic, alone or in combination with zinc. However, sleep quality was influenced only by probiotic and zinc combination.

**Trail Registration:** This study has been registered in the Iranian registry of clinical trials (IRCT20170609034406N9).

## Graphical abstract

Health‐related quality of life improved with probiotic and its combination with zinc. Sleep quality was improved by probiotic and zinc combination. Treatments had no effect on overt hepatic encephalopathy recurrence, ammonia levels, and clinical score.

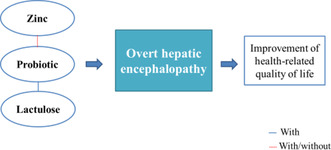

**Abbreviations**
ANCOVA: analysis of covariance
ANOVA: one‐way analysis of variance
HE: hepatic encephalopathy
HRQoL: health‐related quality of life
LAC: lactulose
MELD: model for end‐stage liver disease
MHE: minimal HE
OHE: overt HE
PROB: probiotic
PSQI: Pittsburgh sleep quality index
RCT: randomized controlled clinical trial
RE: emotional health
RP: physical health
SF‐36: short form health survey
Zn: zinc

## 1 Introduction

Hepatic encephalopathy (HE) is a reversible neuropsychiatric syndrome and a common complication of advanced liver diseases. It comprises a spectrum of disorders ranging from sleep–wake inversion and mild cognitive alterations to coma and death. HE is linked to the brain accumulation of ammonia following hepatic clearance dysfunction or portosystemic shunting (Mandiga, Foris, and Bollu 2022). Ammonia, a by‐product of nitrogen metabolism, is predominantly produced by enterocytes and colonic bacteria (Ferenci 2017). Therefore, the gut microbiome and its alteration by antibiotics, probiotics, or prebiotics play fundamental roles in the pathogenesis or management of disease (Khungar and Poordad 2012). HE is associated with low quality of life, poor survival, and a high economic burden on family and society (Bajaj 2010).

Overt HE (OHE), clinically manifested HE, is estimated to occur in around 30%–45% of patients with cirrhosis (Bajaj 2010). OHE management is dependent on the underlying causes, including renal failure, infection, dehydration, gastrointestinal bleeding, excessive dietary protein intake, medication non‐compliance, and so forth. (Mandiga, Foris, and Bollu 2022). Lactulose is a non‐absorbable disaccharide identified as the first‐line agent for HE management (Gluud, Vilstrup, and Morgan 2016). Several mechanisms have been proposed to explain the beneficial effects of this prebiotic on the occurrence and recurrence of HE (Sharma et al. 2012; Agrawal et al. 2012). These mechanisms include: modifying colonic flora by replacing urease‐producing bacteria with non‐urease‐producing ones; promoting colonic ammonia uptake by bacteria for the production of nitrogenous compounds; and increasing hyperosmolar loading in the colon, which enhances gastrointestinal motility, reduces ammonia absorption, and facilitates its excretion (Mukherjee and John 2022). Nonetheless, poor adherence to lactulose therapy due to its unpleasant taste and side effects, including diarrhea, nausea, bloating, flatulence, and vomiting, are reported. Dehydration and electrolyte disturbances occur following severe diarrhea and vomiting, which deteriorate HE (Khungar and Poordad 2012). Therefore, many researchers are seeking a safer and more effective agent for HE prevention or treatment to replace or combine with lactulose.

Recent studies have focused on the efficacy of probiotics or synbiotics (probiotics and prebiotics) on the HE in cirrhosis. A decreased count of Lactobacillus and Bifidobacterium genera is detected in the gut microbiome of patients suffered from cirrhosis (Huang et al. 2022). The VSL#3 probiotic, which is a combination of *Lactobacillus plantarum* , *Lactobacillus paracasei*, *Lactobacillus acidophilus* , *Lactobacillus bulgaricus* , *Bifidobacterium infantis* , *Bifidobacterium longum* , *Bifidobacterium breve* , and *Streptococcus thermophiles*, has been reported to decrease ammonia levels and ameliorate minimal HE (MHE), similar to lactulose (Pratap Mouli et al. 2015). In another trial, VSL#3 supplementation reduced the risk of OHE recurrence but did not affect the ammonia levels (Dhiman et al. 2014).

Zinc is an essential cofactor of ornithine transcarbamylase and glutamine synthetase enzymes, which detoxify ammonia in the body (Nishikawa, Asai, and Fukunishi 2022). Zinc deficiency is usually more prevalent in patients with cirrhosis partly due to poor dietary intakes, alterations in protein and amino acid metabolism, portosystemic shunts, and increased urinary losses (Gruengreiff, Reinhold, and Wedemeyer 2016). Zinc supplementation improved neuropsychometric tests and quality of life (Janyajirawong, Vilaichone, and Sethasine 2021) but did not affect HE recurrence and ammonia levels in patients with cirrhosis (Shen et al. 2019; Chavez‐Tapia et al. 2013). Nonetheless, evidence is insufficient to conclude whether or not zinc has beneficial effects on the HE conditions.

Due to the limited and conflicting evidence, the present randomized controlled clinical trial (RCT) aimed to assess the efficacy of adding probiotic, alone and in combination with zinc, to routine lactulose therapy on the recurrence of HE, health‐related quality of life (HRQoL), and sleep quality in the patients with cirrhosis.

## 2 Materials and Methods

### 2.1 Study Design

The current prospective RCT was carried out between September 2021 and August 2022 on eligible patients with cirrhosis referring to the Gastroenterology clinics of Imam Reza Hospital, Tabriz, Iran. The protocol of this study was approved by the ethics committee of Tabriz University of Medical Sciences (IR.TBZMED.REC.1399.1144) and registered in the Iranian registry of clinical trials (IRCT20170609034406N9). The research was conducted in accordance with the Helsinki Declaration. All patients signed written informed consent form before commencement of the study.

### 2.2 Participants

Patients between 18 and 80 years‐old with decompensated cirrhosis, according to the diagnostic criteria defined by the gastroenterologists and Model for End‐stage Liver Disease (MELD) score > 10, and with a previous history of OHE were included in the study. The exclusion criteria were as follows: pregnancy or breastfeeding, history of probiotic or zinc consumption in the last 6 weeks, history of OHE within previous 6 months, alcohol drinking 6 weeks prior to or during the intervention, non‐adherence to the interventions or dietary recommendations, taking psychotropic drugs (selective serotonin reuptake inhibitors, serotonin and norepinephrine reuptake inhibitors, tricyclic antidepressant, benzodiazepines, barbiturates, narcotic drugs, gamma aminobutyric acid analogues, etc.), suffering from neurological disorders (Parkinson's, Alzheimer's, and non‐hepatic metabolic encephalopathy), severe infections, and cancer as well as a previous history of liver surgery or transjugular intrahepatic portosystemic shunt (TIPS) procedure.

### 2.3 Randomization and Intervention

Eligible patients were allocated randomly into three equal groups using the permuted block randomization technique with random block sizes of 6 and 9 by random sequence generator software. The randomization sequence was concealed using sealed opaque envelopes with consecutive numbering. The eligible patients were assigned to receive lactulose syrup (LAC group and Control), lactulose and probiotic (LAC‐PROB group), or a combination of lactulose, probiotic, and zinc (LAC‐PROB‐Zn group) for 3 months. All patients received 30–45 mL lactulose syrup, 2–4 times/day, to achieve at least two soft stools/day (according to the drug monograph). Patients in the LAC‐PROB and LAC‐PROB‐Zn groups also were treated with two probiotic capsules (Comflor, Farabiotic, Iran) per day; each capsule contained 4.5 × 10^11^ colony‐forming units (CFU) of bacteria, similar to the VSL#3 (a combination of *Lactobacillus plantarum* , *Lactobacillus paracasei*, *Lactobacillus acidophilus* , *Lactobacillus bulgaricus* , *Bifidobacterium infantis* , *Bifidobacterium longum* , *Bifidobacterium breve* , and *Streptococcus thermophiles*). One 25 mg zinc gluconate capsule (Alhavi Pharmaceutical Company, Iran) also was given daily to patients of the LAC‐PROB‐Zn group.

### 2.4 Study Procedures and Outcomes

At first, a general questionnaire, including sex, age, and medical and drug history, was completed for eligible participants. The HRQoL of the participants and their sleep quality were also assessed by the short form health survey (SF‐36) (Ware Jr and Sherbourne 1992) and the Pittsburgh Sleep Quality Index (PSQI) (Buysse et al. 1989) questionnaires, respectively. The validity and reliability of the Persian version of these questionnaires have been confirmed previously (Farrahi Moghaddam et al. 2012; Montazeri et al. 2005). The SF‐36 questionnaire evaluates eight different domains, including physical functioning, role limitation caused by physical health (RP), bodily pain, general health, vitality, social functioning, role limitation caused by emotional health (RE), and mental health, resulting in a physical or mental component summary. A higher score of SF‐36 indicates better HRQoL of participants. The PSQI questionnaire also evaluates seven areas of sleep, including subjective sleep quality, sleep latency, sleep duration, habitual sleep efficiency, sleep disturbances, use of sleeping medication, and daytime dysfunction. A total score of PSQI ≥ 5 denotes poor sleeping quality.

Blood samples were collected from the patients to assess their bilirubin, creatinine, and prothrombin time. Ammonia levels also were evaluated at the same time by collecting venous blood samples from the patients without using a tourniquet. The samples were then immediately transferred to an Ethylenediamine tetraacetic acid (EDTA) tube and centrifuged. The concentration of ammonia was measured by measuring the absorption through a spectrophotometer after 30 s and 2.5 min. The final ammonia concentration was calculated by dividing the difference in absorption after 2.5 min and 30 s by the difference in absorbance of standard and multiplying it by the standard's concentration (Bajaj et al. 2020).

All patients were advised verbally to follow dietary recommendations for cirrhosis, including eating low‐sodium and frequent small‐size meals; reducing trans and saturated fat intake; and consuming whole grains, low‐fat dairy, legumes, and soybean (Mahan and Raymond 2021). Besides, they were asked not to consume probiotic dairy and zinc‐containing supplements during the study. A sheet containing these recommendations was also delivered to all participants at the baseline. Then, patients were randomly allocated into three groups to receive the treatments for 3 months. Telephonic reminders were given to patients every 2 weeks for following medication compliance. The patients were visited every month by their physicians, and medication compliance was assessed by considering the remaining capsules or syrup volume and interviews with the patients. 12 weeks after study enrollment, SF‐36 and PSQI questionnaires were completed again for each patient, and blood samples were obtained.

### 2.5 Sample Size Calculation and Statistical Analysis

Due to the lack of similar studies in the literature, we conducted a pilot study to determine the appropriate sample size for the trial. A pilot sample consisting of four participants per group was selected to provide preliminary data on the variability of outcomes (specifically PSQI). Although small, this sample allowed us to estimate the key parameters needed for formal sample size calculation in the main trial. Pilot studies are commonly used to assess feasibility and to gather early insights when no prior data are available, as supported by literature (Hertzog 2008; Julious 2005). Based on the pilot data, we then calculated the final sample size required for adequate statistical power in the full study. Given *α* = 0.05 and *β* = 0.2 and using G power 3.1.9.2 software, the sample size in each group was determined to be 14, which was increased to 15 (total patients: 45).

Data were analyzed using SPSS version 26. The normality of the quantitative data was assessed via the Smirnov–Kolmogorov test. The quantitative and qualitative variables are presented as mean ± SD and number (percentage), respectively. Differences between the groups were evaluated using one‐way analysis of variance (ANOVA) for parametric and Chi‐squared test for nominal variables. Analysis of covariance (ANCOVA) was used to compare the post‐treatment values of quantitative variables between groups by adjusting for related baseline data and confounding factors. The Bonferroni post hoc test was conducted when significant differences were observed. Furthermore, a paired samples *t*‐test was performed to assess within‐group changes. *p* < 0.05 was considered significant.

## 3 Results

**FIGURE 1:**
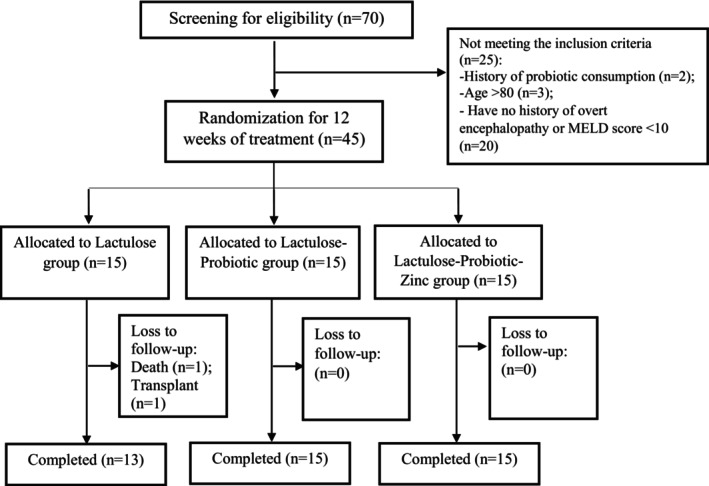
CONSORT diagram.

Seventy consecutive patients with cirrhosis were assessed regarding our eligibility criteria. Of these, 45 patients met our inclusion criteria and were included. Two patients in the LAC group withdrew from the study due to death and liver transplant, respectively. Finally, 43 subjects completed the follow‐up (Figure 1). The strict compliance to the study protocol (100%) was documented in the present study. Patients in the control group experienced known side effects associated with lactulose, including diarrhea (20%), flatulence, and unpleasant taste (10%), and zinc and probiotic did not induce additional side effects.

### 3.1 Patients' Characteristics

Baseline characteristics of the included patients in the different groups are presented in Table 1. The mean age of the total participants in our study was 47.62 (minimum: 21, maximum: 70) years with male preponderance (57.8%). Autoimmune hepatitis (22.2%) and hepatitis B virus (11.1%) were the most prevalent etiology of cirrhosis in the included subjects. There were no significant differences in age (*p* = 0.399), sex (*p* = 0.802), and etiology (*p* = 0.937) distribution between three groups.

**TABLE 1:** Baseline characteristics of the patients.

Variable	LAC Group (*n* = 15)	LAC‐PROB Group (*n* = 15)	LAC‐PROB‐Zn Group (*n* = 15)	*p*
Age (year), mean ± SD	48.20 ± 14.05	50.67 ± 14.24	44.00 ± 12.04	0.399
Sex, *n* (%)				
Male	10 (66.7)	8 (53.3)	8 (53.3)	0.802
Female	5 (33.3)	7 (46.7)	7 (46.7)	
Cirrhosis etiology, *n* (%)				
NASH	1 (6.7)	0 (0.0)	1 (6.7)	0.937
AIH	2 (13.3)	4 (26.7)	4 (26.7)	
HBV	1 (6.7)	2 (13.3)	2 (13.3)	
HCV	2 (13.3)	1 (6.7)	0 (0.0)	
PSC	1 (6.7)	2 (13.3)	1 (6.7)	
Cryptogenic	8 (53.3)	6 (40.0)	7 (46.7)	

*Note:* Age variable was statistically analyzed by one‐way analysis of variance (ANOVA) and the other variables using Fisher's exact test. *p* < 0.05 were considered as significant.

Abbreviations: AIH: autoimmune hepatitis, HBV: hepatitis B virus, HCV: hepatitis C virus, LAC: lactulose, NASH: nonalcoholic steatohepatitis, PSC: primary sclerosing cholangitis, PROB: probiotic, Zn: zinc.

### 3.2 Recurrence of OHE

OHE was developed only in two patients of the LAC group during the trial. None of the participants in the intervention groups experienced OHE recurrence. There was no significant difference in the OHE recurrence between three groups (*p* = 0.318).

### 3.3 Ammonia Levels and MELD Score

As shown in Table 2, there were no significant differences in the pre‐ and post‐intervention levels of ammonia and MELD score between examined groups. The adjustment for baseline variables, age, and sex did not change the results. The within‐group analysis revealed the reduced MELD score only in the LAC‐PROB group (mean change = −1.00 ± 1.51, *p* = 0.023).

**TABLE 2:** Comparison of plasma ammonia values and MELD Score at the baseline and after 12 weeks of intervention between different groups.

Variable	LAC Group	LAC‐PROB Group	LAC‐PROB‐Zn Group	*p* (between)**	Adjusted *p* (between)***
Ammonia (μmol/L)					
Before	75.45 ± 33.22	64.54 ± 32.18	56.91 ± 34.55	0.319	0.465
After	68.92 ± 31.36	52.63 ± 20.08	58.17 ± 40.27	0.399	
Mean change	−2.96 (−31.05 to 25.13)	−11.91 (−32.29 to 8.48)	−8.58 (−18.55 to 1.37)	0.509	
*p* (within)*	0.822	0.231	0.908		
MELD score					
Before	15.07 ± 4.43	13.93 ± 2.25	14.33 ± 2.66	0.630	0.268
After	14.23 ± 2.95	12.93 ± 2.12	13.67 ± 3.64	0.515	
Mean change	0.31 (−1.18 to 1.80)	−1.00 (−1.84 to −0.16)	−0.67 (−1.75 to 0.41)	0.127	
*p* (within)*	0.660	**0.023**	0.207		

*Note:* Data are expressed as mean ± standard deviation or mean change (95%CI). *p* < 0.05 were considered as significant. Bold type denotes significance.

Abbreviations: CI: confidence interval, LAC: lactulose, MELD: model for end‐stage liver disease, PROB: probiotic, Zn: zinc.

* *P*‐value based on paired samples *t*‐test.

** *P*‐value based on one‐way analysis of variance (ANOVA).

*** *P*‐value based on analysis of covariance (ANCOVA) adjusted for baseline measures, age, and sex.

### 3.4 HRQoL

After 3 months, the SF‐36 total scores were raised by 26.5% and 23.8% in the LAC‐PROB and LAC‐PROB‐Zn groups, respectively, which were significantly higher than the control group. Mental health and mental component summary were also significantly improved in the LAC‐PROB and LAC‐PROB‐Zn groups compared to the LAC group, even after adjustment for baseline values, age, and sex. However, RP, bodily pain, general health, vitality, social functioning, and physical component summary were only increased by LAC‐PROB‐Zn compared to the lactulose alone. The effect of LAC‐PROB‐Zn on the RP and physical component summary were significantly stronger than the LAC‐PROB (Table 3). We also observed that one patient in the control group (7.7%), 11 in the LAC‐PROB group (73.3%), and 12 in the LAC‐PROB‐Zn group (80%) met or exceeded the minimal clinically important difference (MCID) (4.6) (Clement, Weir, and Deehan 2022) for a total score of SF‐36.

**TABLE 3:** Within and between group analysis of short form health survey (SF‐36) parameters.

SF‐36 Parameters	LAC Group	LAC‐PROB Group	LAC‐PROB‐Zn Group	*p* (between)**	Adjusted *p* (between)***
Physical functioning					
Before	65.67 ± 20.52	51.33 ± 30.85	73.00 ± 21.86	0.063	
After	67.92 ± 16.71	59.67 ± 30.09	75.67 ± 19.17	0.180	0.405
Mean change	−3.08 (−7.94 to 1.79)	8.33 (−1.41 to 18.08)	2.67 (−6.51 to 11.84)	0.145	
*p* (within)*	0.309	0.088	0.543		
Role limitation caused by physical health					
Before	53.33 ± 35.19	35.00 ± 43.09	43.33 ± 45.77	0.488	
After	55.77 ± 32.52^a^	40.00 ± 46.10^a^	78.33 ± 31.15^b^	**0.027**	**< 0.001**
Mean change	−3.85 (−15.94 to 8.25)^a^	5.00 (−17.93 to 27.93)^a^	35.00 (14.87 to 55.13)^b^	**0.011**	
*p* (within)*	0.502	0.647	**0.002**		
Bodily pain					
Before	73.50 ± 18.15	52.00 ± 24.70	59.00 ± 30.72	0.068	
After	71.73 ± 24.29^a^	76.33 ± 23.07^ab^	89.83 ± 12.76^b^	0.060	**0.008**
Mean change	−3.65 (−14.04 to 6.73)^a^	24.33 (12.84 to 35.83)^b^	30.83 (13.81 to 47.85)^b^	**0.001**	
*p* (within)*	0.458	**< 0.001**	**0.002**		
General health					
Before	50.00 ± 17.52	48.67 ± 19.41	60.68 ± 22.27	0.203	
After	46.61 ± 16.17^a^	61.00 ± 19.38^ab^	70.67 ± 21.29^b^	**0.008**	**0.015**
Mean change	−5.31 (−13.40 to 2.79)	12.33 (0.50 to 24.17)	9.99 (−2.62 to 22.59)	0.053	
*p* (within)*	0.179	**0.042**	0.111		
Vitality					
Before	50.33 ± 17.16	42.33 ± 27.25	63.33 ± 27.17	0.069	
After	48.85 ± 12.77^a^	57.25 ± 22.84^ab^	72.00 ± 17.30^b^	**0.006**	**0.016**
Mean change	−4.61 (−14.52 to 5.29)	14.92 (3.33 to 26.50)	8.67 (−7.63 to 25.10)	0.093	
*p* (within)*	0.330	**0.015**	0.277		
Social functioning					
Before	67.50 ± 25.35	54.17 ± 31.58	71.67 ± 25.65	0.207	
After	65.38 ± 18.51^a^	62.50 ± 27.14^ab^	85.00 ± 19.02^b^	**0.017**	**0.033**
Mean change	−4.81 (−14.33 to 4.72)	8.33 (−5.67 to 22.34)	8.67 (−7.76 to 25.10)	0.277	
*p* (within)*	0.293	0.223	0.131		
Role limitation caused by emotional health					
Before	28.89 ± 39.57	48.90 ± 46.91	57.77 ± 40.76	0.175	
After	30.77 ± 39.59	51.11 ± 50.18	80.00 ± 30.35	**0.010**	**0.045**
Mean change	2.57 (−7.37 to 12.50)	2.22 (−19.24 to 23.69)	22.22 (2.90 to 41.54)	0.169	
*p* (within)*	0.584	0.827	**0.027**		
Mental health					
Before	62.49 ± 16.33	46.63 ± 20.04	69.72 ± 16.12	**0.003**	
After	58.58 ± 10.80^a^	68.69 ± 19.83^b^	75.90 ± 15.60^b^	**0.028**	**0.005**
Mean change	−5.39 (−15.73 to 4.95)^a^	22.06 (13.05 to 31.07)^b^	6.61 (−2.11 to 15.33)^a^	**< 0.001**	
*p* (within)*	0.278	**< 0.001**	0.209		
Physical component summary					
Before	58.23 ± 17.16	45.87 ± 23.11	59.87 ± 21.38	0.141	
After	57.82 ± 14.32^a^	58.85 ± 19.01^a^	77.30 ± 14.73^b^	**0.003**	**< 0.001**
Mean change	−3.60 (−8.82 to 1.62)^a^	12.98 (3.06 to 22.91)^b^	17.43 (9.18 to 25.68)^b^	**0.001**	
*p* (within)*	0.163	**0.014**	**< 0.001**		
Mental component summary					
Before	51.84 ± 16.99	48.14 ± 24.29	64.63 ± 18.25	0.073	
After	50.04 ± 12.96^a^	60.11 ± 22.21^b^	76.80 ± 15.56^b^	**0.001**	**0.001**
Mean change	−3.51 (−9.69 to 2.67)^a^	11.97 (1.46 to 22.48)^b^	12.16 (3.86 to 20.47)^b^	**0.015**	
*p* (within)*	0.239	**0.028**	**0.007**		
Total score of SF‐36					
Before	55.04 ± 15.91	47.00 ± 23.15	62.25 ± 17.67	0.105	
After	53.93 ± 12.46^a^	59.48 ± 18.97^b^	77.05 ± 14.17^b^	**0.001**	**< 0.001**
Mean change	−3.54 (−8.97 to 1.89)^a^	12.48 (3.38 to 21.58)^b^	14.80 (7.64 to 21.96)^b^	**0.002**	
*p* (within)*	0.181	**0.011**	**0.001**		

*Note:* Data are expressed as mean ± standard deviation or mean change (95%CI). *p* < 0.05 were considered as significant. Bold type denotes significance. Different letters indicate significant differences for the adjusted model and mean changes.

Abbreviations: CI: confidence interval, LAC: lactulose, PROB: probiotic, Zn: zinc.

* *P*‐value based on paired samples *t*‐test.

** *P*‐value based on one‐way analysis of variance (ANOVA).

*** *P*‐value based on analysis of covariance (ANCOVA) adjusted for baseline measures, age, and sex. The Bonferroni post hoc test was conducted when significant differences were observed.

### 3.5 Sleep Quality and Disturbances

As shown in Table 4, the LAC‐PROB‐Zn group had better subjective sleep quality and total score of PSQI than the control and LAC‐PROB groups. These results remained unchanged after adjustment for baseline values, age, and sex. Following adjustment, we detected an improvement in sleep duration in the patients receiving LAC‐PROB‐Zn compared to the LAC (*p* = 0.037). However, data related to sleep disturbances became non‐significant.

**TABLE 4:** Within and between group analysis of Pittsburgh Sleep Quality Index (PSQI) score.

PSQI Parameters	LAC Group	LAC‐PROB Group	LAC‐PROB‐Zn Group	*p* (Between)**	Adjusted *p* (Between)***
Subjective sleep quality					
Before	1.47 ± 0.83	1.07 ± 1.16	0.73 ± 0.70	0.104	
After	1.31 ± 0.63^a^	1.13 ± 1.06^a^	0.20 ± 0.41^b^	**0.001**	**0.002**
Mean change	−0.08 (−0.24 to 0.09)	0.07 (−0.38 to 0.51)	−0.53 (−0.94 to −0.12)	**0.046**	
*p* (within)*	0.337	0.751	**0.015**		
Sleep latency					
Before	1.80 ± 0.86	1.87 ± 1.06	1.87 ± 1.30	0.981	
After	1.92 ± 1.04	1.80 ± 1.15	1.20 ± 0.94	0.152	0.089
Mean change	0.08 (−0.31 to 0.46)	−0.07 (−0.60 to 0.46)	−0.67 (−1.35 to 0.017)	0.115	
*p* (within)*	0.673	0.792	0.055		
Sleep duration					
Before	1.13 ± 0.83	1.40 ± 1.30	1.07 ± 1.10	0.680	
After	1.15 ± 0.90^a^	1.47 ± 1.19^ab^	0.60 ± 0.83^b^	0.064	**0.018**
Mean change	0.15 (−0.33 to 0.64)^a^	0.07 (−0.26 to 0.39)^ab^	−0.47 (−0.75 to −0.18)^b^	**0.026**	
*p* (within)*	0.502	0.670	**0.004**		
Habitual sleep efficiency					
Before	0.47 ± 0.91	1.13 ± 1.30	0.93 ± 1.10	0.256	
After	0.38 ± 0.51	1.13 ± 1.19	0.53 ± 0.74	0.063	0.232
Mean change	0.23 (−0.03 to 0.49)	0.00 (−0.51 to 0.51)	−0.40 (−0.94 to 0.14)	0.140	
*p* (within)*	0.082	1.00	0.138		
Sleep disturbances					
Before	1.60 ± 0.51	1.53 ± 0.52	1.13 ± 0.52	**0.035**	
After	1.38 ± 0.51	1.47 ± 0.52	0.93 ± 0.26	**0.004**	0.074
Mean change	−0.15 (−0.49 to 0.18)	−0.07 (−0.32 to 0.19)	−0.20 (−0.43 to 0.03)	0.740	
*p* (within)*	0.337	0.582	0.082		
Use of sleeping medication					
Before	0.00 ± 0.00	0.00 ± 0.00	0.00 ± 0.00	ns	
After	0.00 ± 0.00	0.00 ± 0.00	0.00 ± 0.00	ns	ns
Mean change	0.00 ± 0.00	0.00 ± 0.00	0.00 ± 0.00		
*p* (within)*	Ns	ns	ns		
Daytime dysfunction					
Before	0.73 ± 0.88	1.13 ± 1.25	0.73 ± 1.03	0.499	
After	0.77 ± 0.60	0.47 ± 0.99	0.53 ± 0.83	0.612	0.248
Mean change	0.23 (−0.27 to 0.73)	−0.67 (−1.21 to −0.13)	−0.20 (0.80 to 0.40)	0.063	
*p* (within)*	0.337	**0.019**	0.486		
Total score					
Before	7.20 ± 3.00	8.13 ± 5.26	6.47 ± 4.21	0.566	
After	6.92 ± 2.29^a^	7.53 ± 4.72^a^	4.00 ± 2.62^b^	**0.017**	**0.001**
Mean change	0.46 (−0.22 to 1.14)^a^	−0.60 (−1.97 to 0.77)^ab^	−2.47 (−4.03 to −0.90)^b^	**0.006**	
*p* (within)*	0.165	0.363	**0.004**		
Poor sleeping quality (total score ≥ 5)					
Before	13 (86.7)	11 (73.3)	10 (66.7)	0.431^****^	
After	12 (92.3)	10 (66.7)	7 (46.7)	**0.037** ^****^	

*Note:* Poor sleeping quality is reported as frequency (percentage) and other data are expressed as mean ± standard deviation or mean change (95%CI). *p* < 0.05 were considered as significant. Bold type denotes significance. Different letters indicate significant differences for the adjusted model and mean changes.

Abbreviations: CI: confidence interval, LAC: lactulose, PROB: probiotic, Zn: zinc.

* *P*‐value based on paired samples *t*‐test.

** *P*‐value based on one‐way analysis of variance (ANOVA).

*** *P*‐value based on analysis of covariance (ANCOVA) adjusted for baseline measures, age, and sex.^****^ *P*‐value based on the Chi‐squared test. The Bonferroni post hoc test was conducted when significant differences were observed.

We also found that eight patients in the LAC‐PROB‐Zn group (53.3%), two in the LAC‐PROB group (13.3%), and none in the control group experienced a clinically significant improvement in sleep quality (PSQI total score reduction ≥ 3 (Eadie et al. 2013; Costandi et al. 2022)), compared to the baseline.

## 4 Discussion

The results of our RCT revealed no effect of interventions on the OHE recurrence, ammonia level, and MELD score compared to the lactulose alone. Our findings were in line with some previous studies. According to a meta‐analysis by Xu et al., probiotics had no effects on serum ammonia in patients with cirrhosis (Xu et al. 2014). In another meta‐analysis of two RCTs (*n* = 137), no effect of the zinc and lactulose combination on serum ammonia levels, compared to the lactulose alone, was detected in patients with cirrhosis (Shen et al. 2019). Furthermore, zinc supplementation did not affect 1‐month or 1‐year all‐cause or OHE‐dependent readmission rates, ammonia levels, and MELD scores in cirrhotic patients complicated by HE (Janyajirawong, Vilaichone, and Sethasine 2021; Fritz et al. 2022). Contrary to our results, a body of literature outlines the beneficial effects of probiotics or zinc on the HE and hyperammonemia condition in patients with cirrhosis (Pratap Mouli et al. 2015; Takuma et al. 2010). In a previous RCT, patients with MHE were randomized to lactulose or VSL#3 probiotic (4.5 × 10^11^ CFU/day) capsules for 2 months. The results showed a similar effect of VSL#3 probiotic relative to lactulose on improving MHE and reducing ammonia levels (Pratap Mouli et al. 2015). In another double‐blind RCT, 6 months of VSL#3 (9 × 10^11^ bacteria) supplementation reduced the risk of OHE recurrence, risk of hospitalization for OHE, and MELD and Child–Turcotte–Pugh scores, but not the ammonia level, in the cirrhotic patients who had recovered from a previous episode of OHE (Dhiman et al. 2014). This contradictory evidence could be due to the differences in the study sample, dose and duration of interventions, and clinical stage of the disease.

Cirrhosis, especially in advanced stages, is linked to decreased HRQoL (Orr et al. 2014). It is recommended that physicians focus not only on clinical features but also on patient‐reported outcomes, like the quality of life and well‐being (Younossi and Henry 2015). The HRQoL of our studied population was higher than the reported values in other Iranian patients suffered from cirrhosis (Mohamadnejad et al. 2007; Nouri‐Vaskeh et al. 2020). In the present study, we also observed improved mental and total quality of life following probiotics and also probiotics and zinc combination. Besides, most domains of HRQoL, excluding physical functioning and RE, were improved only by LAC‐PROB‐Zn. Our findings were in line with some previous studies. Janyajirawong et al. reported that all domains of Sf‐36, except for bodily pain and vitality, became better, compared to the placebo, following 12 weeks of zinc therapy in patients with MHE (Janyajirawong, Vilaichone, and Sethasine 2021). In another study, VSL#3 supplementation for 6 months improved physical functioning, RP, and physical component summary in patients with cirrhosis compared to the before (Dhiman et al. 2014). Nonetheless, a combination of *Streptococcus thermophilus* , *Lactobacillus bulgaricus* , *Lactobacillus acidophilus* , *Lactobacillus casei* , and *Bifidobacteria* for 60 days did not impact on SF‐36 physical and mental functioning compared to the placebo in MHE patients (Bajaj et al. 2008). Therefore, more studies should be performed to clarify these effects.

Many cirrhotic patients, especially after HE occurrence, experience sleep disturbances, including insomnia and daytime sleepiness. Poor sleep could influence on patient's quality of life and mortality (Zhao and Wong 2016). In this study, only patients who received lactulose–probiotic–zinc combination reported an improved sleep quality compared to the others. It is worth mentioning that when we excluded nine patients who were > 60 years old, the LAC‐PROB‐Zn group had still significantly better total SF‐36 and PSQI scores compared to the both control and LAC‐PROB groups. Similar to our results, K. Dhiman et al. demonstrated no effect of oral administration of 9 × 10^11^ CFU/day VSL#3 on the PSQI in patients with cirrhosis (Dhiman et al. 2014). In another RCT, 1 month of 220 mg zinc sulfate supplementation every 3 days improved subjective sleep quality and the total score of PSQI in the nurses (Gholipour Baradari et al. 2018). Furthermore, a positive correlation between serum zinc levels and sleep quality was detected in patients with advanced cirrhosis (Nishikawa et al. 2020). However, no previous study has assessed the effects of zinc supplementation, alone or in combination with probiotics, on sleep disturbances in patients with cirrhosis.

Our study was the first to examine the effects of probiotics and zinc combination on patients with cirrhosis. We used standard questionnaires and participants were not connected with each other and completed the surveys independently. This study had several limitations. First, we could not use psychometric tests, as planned in our protocol, because most included patients were illiterate or low‐educated, and there was no valid Persian version of the tests. Second, the study duration was probably short to expect significant changes in some parameters like OHE recurrence, and the sample size was small for conducting subgroup analysis. Thirdly, patients were not blinded to the interventions. Fourthly, our study did not include a separate group receiving lactulose and zinc therapy, which could be a valuable consideration for future research to enhance the comprehensiveness of findings.

In conclusion, adding probiotic, alone or together with zinc, to lactulose for 3 months exerts no effect on OHE recurrence, ammonia levels, and clinical score but improves HRQoL in patients with cirrhosis. Furthermore, a combination of lactulose, probiotic, and zinc improves sleep quality in these patients. More well‐designed long‐duration RCTs are needed to clarify the effects of probiotics and zinc on cirrhosis.

## Author Contributions

**Leila Amooyi:** conceptualization (lead), data curation (equal), formal analysis (equal), investigation (lead), methodology (lead), software (equal), writing – original draft (equal). **Leila Alizadeh:** investigation (equal), methodology (equal), writing – review and editing (equal). **Parvin Sarbakhsh:** data curation (equal), formal analysis (lead), software (lead), writing – review and editing (equal). **Sara Shojaei‐Zarghani:** methodology (equal), writing – original draft (equal). **Afshin Gharekhani:** conceptualization (lead), data curation (lead), methodology (lead), supervision (lead), validation (lead), visualization (lead), writing – review and editing (lead).

## Ethics Statement

The protocol of this study was approved by the ethics committee of Tabriz University of Medical Sciences (IR.TBZMED.REC.1399.1144) and registered in the Iranian registry of clinical trials (IRCT20170609034406N9). The research was conducted in accordance with the Helsinki Declaration.

## Consent

All patients signed the written informed consent form before commencement of the study.

## Conflicts of Interest

The authors declare no conflicts of interest.

## Data Availability

Data supporting the results of this study is available from the corresponding author upon reasonable request.
